# Glomerular Collagen Deposition and Lipocalin-2 Expression Are Early Signs of Renal Injury in Prediabetic Obese Rats

**DOI:** 10.3390/ijms20174266

**Published:** 2019-08-30

**Authors:** Eva Nora Bukosza, Tamás Kaucsár, Mária Godó, Enikő Lajtár, Pál Tod, Gábor Koncsos, Zoltán V. Varga, Tamás Baranyai, Nguyen Minh Tu, Helga Schachner, Csaba Sőti, Péter Ferdinandy, Zoltán Giricz, Gábor Szénási, Péter Hamar

**Affiliations:** 1 Institute of Pathophysiology, Semmelweis University, H-1089 Budapest, Hungary; 2 Department of Pathology, Medical University of Vienna, 1090 Wien, Austria; 3 Department of Pharmacology and Pharmacotherapy, Semmelweis University, H-1089 Budapest, Hungary; 4 Department of Medical Chemistry, Molecular Biology and Pathobiochemistry, Semmelweis University, H-1089 Budapest, Hungary; 5 Pharmahungary Group, H-1089 Budapest, Hungary; 6 Institute of Clinical Experimental Research, Semmelweis University, H-1089 Budapest, Hungary; 7 Institute of Translational Medicine, Pécs University, 7624 Pécs, Hungary

**Keywords:** obesity, renal injury, lipocalin-2, collagen type IV, inflammation

## Abstract

Feeding rats with high-fat diet (HFD) with a single streptozotocin (STZ) injection induced obesity, slightly elevated fasting blood glucose and impaired glucose and insulin tolerance, and caused cardiac hypertrophy and mild diastolic dysfunction as published before by Koncsos et al. in 2016. Here we aimed to explore the renal consequences in the same groups of rats. Male Long-Evans rats were fed normal chow (CON; n = 9) or HFD containing 40% lard and were administered STZ at 20 mg/kg (i.p.) at week four (prediabetic rats, PRED, n = 9). At week 21 blood and urine samples were taken and kidney and liver samples were collected for histology, immunohistochemistry and for analysis of gene expression. HFD and STZ increased body weight and visceral adiposity and plasma leptin concentration. Despite hyperleptinemia, plasma C-reactive protein concentration decreased in PRED rats. Immunohistochemistry revealed elevated collagen IV protein expression in the glomeruli, and *Lcn2* mRNA expression increased, while *Il-1β* mRNA expression decreased in both the renal cortex and medulla in PRED vs. CON rats. Kidney histology, urinary protein excretion, plasma creatinine, glomerular Feret diameter, desmin protein expression, and cortical and medullary mRNA expression of *TGF-β1, Nrf2,* and *PPARγ* were similar in CON and PRED rats. Reduced *AMPKα* phosphorylation of the autophagy regulator *Akt* was the first sign of liver damage, while plasma lipid and liver enzyme concentrations were similar. In conclusion, glomerular collagen deposition and increased lipocalin-2 expression were the early signs of kidney injury, while most biomarkers of inflammation, oxidative stress and fibrosis were negative in the kidneys of obese, prediabetic rats with mild heart and liver injury.

## 1. Introduction

Obesity has a worldwide epidemic with a rapidly increasing incidence affecting more than 600 million patients [1]. The burden of obesity is magnified by the various secondary diseases that can develop in obese individuals e.g.,: Non-alcoholic steatohepatitis [2], heart failure with preserved ejection fraction [3], as well as obesity-related glomerulopathy [4,5,6,7]. However, it is unpredictable which disease will develop in a particular patient, and it is also unknown how long the obese state lasts before the first co-morbidity symptoms appear in a patient. Furthermore, many obesity-related diseases can accelerate each other’s progression [5,6], as in the case of hepatorenal syndrome [8]. This complicated picture fuels much research effort to uncover the mechanisms as well as early diagnostic markers of obesity-related co-morbidities.

The detailed mechanisms whereby obesity leads to co-morbidities are far from being understood, but it seems likely that they are initiated by adipose tissue dysfunction. The main characteristics of adipose tissue remodelling are increased production of adipocyte-derived proinflammatory cytokines like leptin, *TNFα*, and *IL-6* [9]. The consequent systemic low-grade inflammation has remote effects on other organs including the kidneys [10]. Locally, lipid accumulation in different organs has been also demonstrated, as a trigger of end-organ damage [11].

We performed a larger project, which evaluated first the general and cardiac effects of high-fat diet (HFD) for 21 weeks with a low dose (20 mg/kg) of streptozotocin (STZ) at week four in Long-Evans rats. According to the results published before, HFD increased body weight and body fat content, and impaired glucose and insulin tolerance in comparison to the control (CON) rats, i.e., the animals became prediabetic (PRED). The interventions caused cardiac hypertrophy and diastolic dysfunction as a possible consequence of cardiac lipid accumulation and dysregulation of mitochondrial fusion and mitophagy in myocytes leading to elevated oxidative stress in the heart. However, no dyslipidemia and blood pressure changes were observed in PRED rats [12]. The aim of the current study was to explore the renal consequences of obesity and prediabetic state in a part of the same rats, in order to reveal early markers of obesity-related renal end-organ damage.

## 2. Results

### 2.1. Twenty Weeks of HFD with a Single Low-Dose of STZ Induced Obesity with Prediabetes, Adipose Tissue Remodelling but Preserved Liver Function in Long-Evans Rats

**Figure 1:**
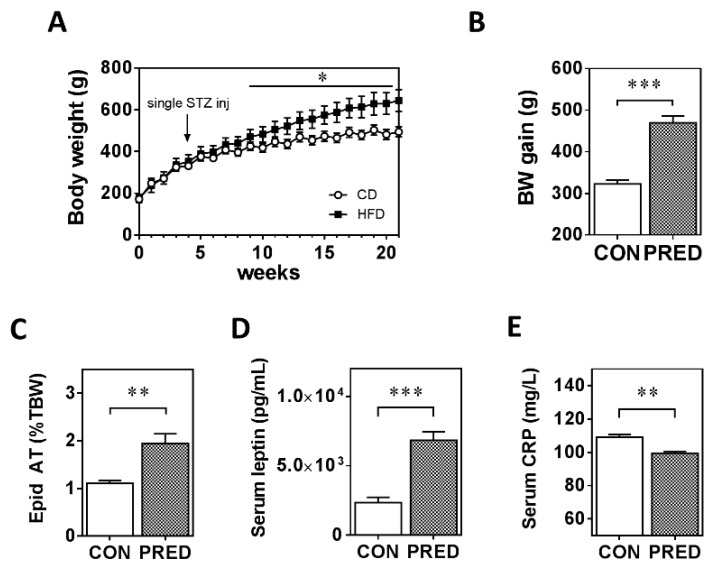
Body weight, plasma and adipose tissue parameters at the end of the study. (**A**) Body weight and (**B**) body weight gain of rats during the study in the two groups, (**C**) epididymal adipose tissue weight (**D**) plasma leptin and (**E**) plasma CRP concentrations in obese prediabetic (PRED, grey columns) and control (CON, white columns) rats at the end of the study. Data are means ± SEM, n ≥ 8/group. Two-way RM ANOVA followed by Sidak’s post hoc test (**A**) and unpaired two-tailed Student’s t-test (B-E); *: *p* < 0.05, **: *p* < 0.01; ***: *p* < 0.0001.

During the 20 weeks follow-up period body weight increased in both groups. However, the body weight of PRED animals was significantly higher, and body weight gain was greater from week 9 compared to the CON group. The difference in body weight between the two groups reached 46% at week 20 (Figure 1A,B). At week 21 the relative epididymal fat tissue weight and plasma leptin levels were significantly higher (Figure 1C,D), while, surprisingly, plasma CRP level was lower in the PRED than in the CON group (Figure 1E).

**Figure 2:**
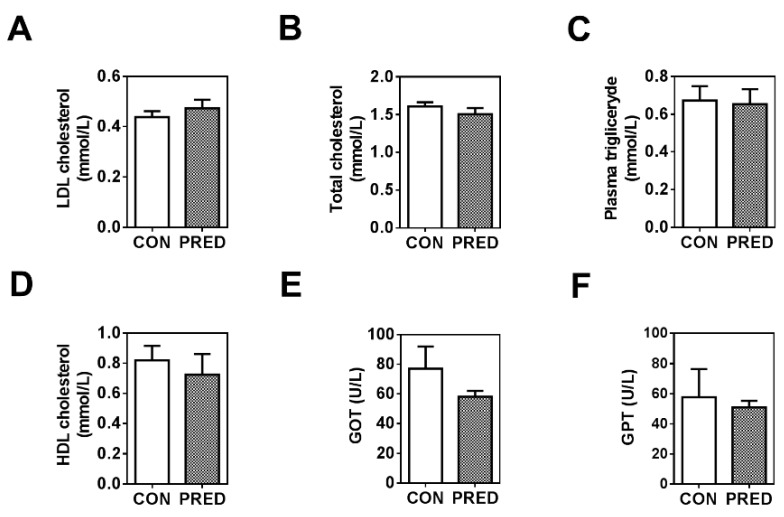
Plasma concentrations of lipids and liver enzymes. (**A**) LDL cholesterol (mmol/L), (**B**) total cholesterol (mmol/L), (**C**) plasma triglyceride (mmol/L), (**D**) HDL cholesterol (mmol/L), (**E**), glutamate oxaloacetate transaminase (GOT; U/L) and (**F**) glutamate pyruvate transaminase (GPT; U/L) concentrations in obese prediabetic (PRED, grey columns) and control (CON, white columns) rats at the end of the study. Data are means ± SEM, n = 11/group. Unpaired two-tailed Student’s *t*-test.

Plasma triglyceride, cholesterol, low density lipoprotein (LDL) and high-density lipoprotein (HDL) levels were similar in the groups (Figure 2A–D). In addition, we could not find any difference in plasma concentrations of liver enzymes (glutamate oxaloacetate transaminase (GOT) and glutamate pyruvate transaminase (GPT)) in the two groups (Figure 2E–F).

**Figure 3:**
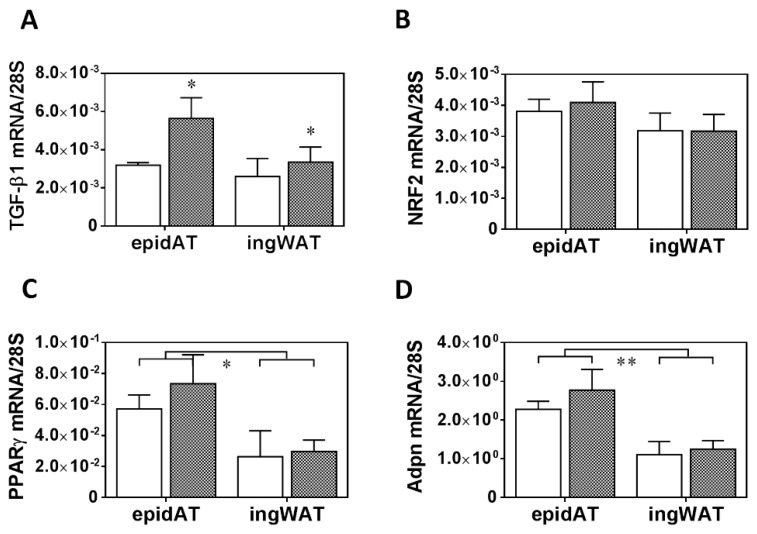
Gene-expression of adipose tissue remodelling indicators. (**A**) *TGF-β1*, (**B**) *Nrf2*, (**C**) *PPARγ*, and (**D**) adiponectin (*Adpn*) mRNA expression in epididymal (epidAT) and inguinal white (ingWAT) adipose tissues in obese prediabetic (PRED, grey columns) and control (CON, white columns) rats at the end of the study. Data are means ± SEM, n ≥ 8/group. Two-way RM ANOVA; *: *p* < 0.05, **: *p* < 0.01; ***: *p* < 0.0001.

*TGF-β1* mRNA expression was higher in epididymal and inguinal adipose tissues in the PRED compared to the CON animals (Figure 3A). On the other hand, *Nrf2*, *PPARγ* and *Adpn* mRNA expression was similar in both fat tissues in the two groups (Figure 3B–D). *PPARγ* and *Adpn* mRNA expression was higher in the epididymal compared to the inguinal white adipose tissue (Figure 3C,D).

### 2.2. Renal Function was Preserved but Significant Glomerular Collagen Deposition and Tubular Lcn2 Expression Appeared in PRED Rats

**Figure 4:**
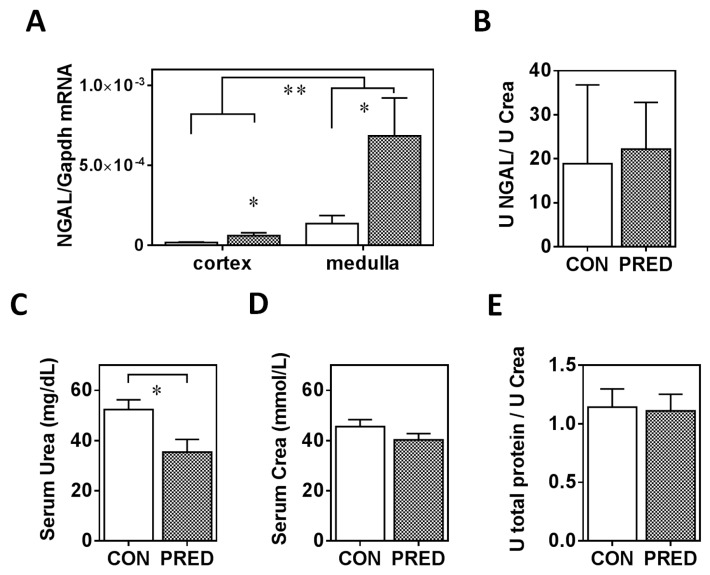
Parameters of renal function and renal injury at the end of the study. (**A**) *Lcn*2 (*NGAL*) mRNA expression in the kidney cortex and medulla, (**B**) urine *Lcn*2 excretion (**C**) plasma urea (mg/dL) and (**D**) plasma creatinine concentrations (mmol/L), (**E**) urinary protein excretion in obese prediabetic (PRED, grey columns) and control (CON, white columns) rats at the end of the study. Data are means ± SEM, n ≥ 7/group two-way RM ANOVA (**A**); and unpaired two-tailed Student’s t-test (B-E); *: *p* < 0.05, **: *p* < 0.01.

Renal function was largely preserved in PRED rats despite obesity and hyperleptinemia. The renal injury marker *Lcn2* (*NGAL*) (mRNA expression) was 7.5-fold and 11.3-fold higher in the medulla and cortex, respectively, in the PRED than in the CON group (Figure 4A). Urine *Lcn2* (*NGAL*) levels were similar in the two groups (Figure 4B). Plasma urea concentration was in the normal range in both groups, but it was significantly lower in the PRED than in the CON group (Figure 4C). Plasma creatinine concentration (Figure 4D) and urinary protein excretion (Figure 4E) were similar in PRED and CON rats.

**Figure 5:**
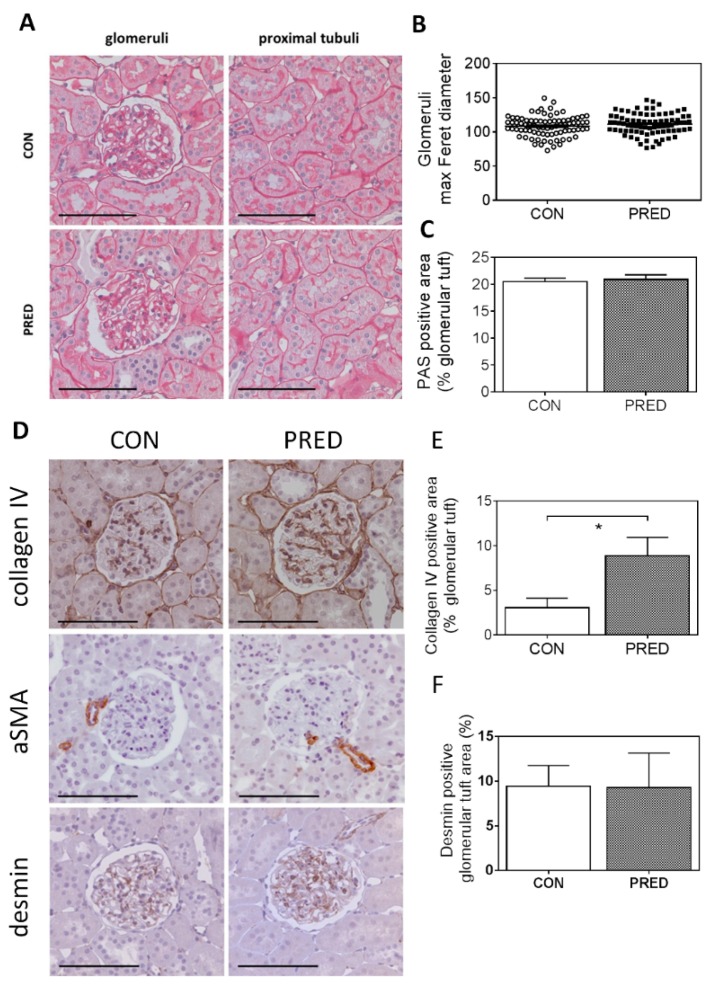
Kidney histology and immunohistochemistry. (**A**) Representative images of periodic-acid-Schiff (PAS) stained glomeruli (left) and proximal tubuli (right) in CON (above) and PRED rats (below). (**B**) The glomerular size indicator maximal Feret diameter; (**C**) PAS positive area relative to total glomerular tuft area. (**D**) Representative images of collagen IV, alpha-smooth muscle actin (αSMA) and desmin immunohistochemistry; (**E**) relative collagen IV positive and (**F**) desmin positive area of the glomerular tuft in control (CON, left) and prediabetic (PRED, right) rats. Photos were taken at 200x magnification; scale bar = 100 μm; data are means ± SEM, n = 9 samples/group, each data point represents mean of 20 glomeruli/samples. Unpaired two-tailed Student’s t-test (**B**,**C**,**E**,**F**); *: *p* < 0.05.

Histological evaluation of PAS stained sections demonstrated intact kidney morphology in both PRED and CON rats (Figure 5A). Average of maximum Feret diameter of glomeruli was similar in the two groups, excluding glomerular hypertrophy in PRED animals (Figure 5B). Relative PAS positive area of the glomerular tuft did not indicate pathologic accumulation of glomerular extracellular matrix (Figure 5C). Brush border of proximal tubular epithelial cells appeared normal and no inverse vacuolar staining was detectable. Oil-red-O staining, a specific marker for lipid accumulation, gave negative results in kidney samples of both the CON and PRED groups despite significant lipid accumulation in liver samples of PRED animals (Supplementary Figure S1).

Collagen IV staining increased significantly in the glomeruli of PRED rats compared to that in CON rats (Figure 5D,E). The mesangial cell dedifferentiation and activation marker, alpha smooth muscle actin (αSMA) protein expression and the podocyte stress indicator desmin protein expression appeared similar in the two groups (Figure 5D–F).

### 2.3. Known mRNA and miRNA Markers of Renal Fibrosis were Unaffected in PRED Rats

**Figure 6:**
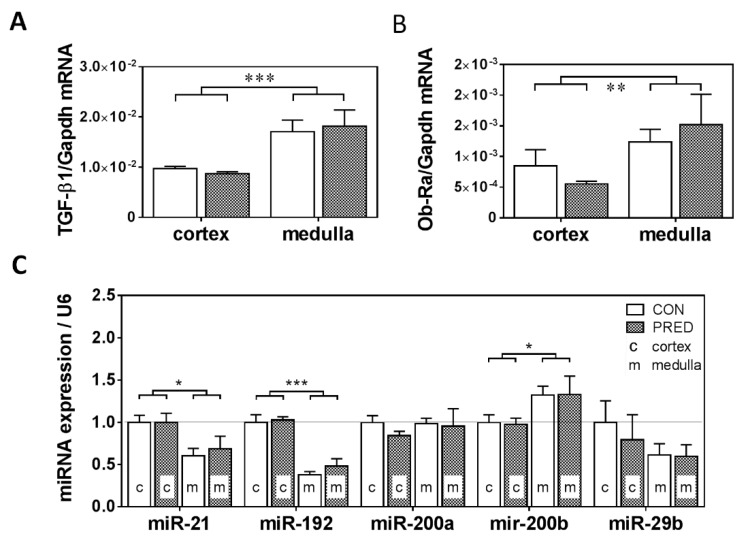
mRNA and miRNA markers of fibrosis in the kidney. (**A**) *TGF-β1* mRNA, (**B**) short-form of leptin-receptor (*Ob-Ra*) mRNA expression and (**C**) expression of renal fibrosis marker microRNAs (miR-21, miR-192, miR-200a, miR-200b, and miR-29b) in the kidney cortex (c) and medulla (m) in obese prediabetic (PRED, gray columns) and control (CON, white columns) rats at the end of the study. Data are means ± SEM, two-way RM ANOVA, n = 8-9/group (**A**–C); n ≥ 4/group, expression of miRNAs is presented relative to their cortical expression in CON (**C**). *: *p* < 0.05, **: *p* < 0.01; ***: *p* < 0.0001.

*TGF-β1* (Figure 6A) and the short-form of leptin-receptor (*Ob-Ra*) mRNAs (Figure 6B) were expressed to the same extent in PRED and CON kidneys. However, there were marked differences in favour of the medullary localization for both mRNAs irrespective of the diet.

Expression of inflammation- and fibrosis-related microRNAs (miR-21, miR-29b, miR-192, miR-200a, miR-200b) in the kidney was not significantly influenced in PRED compared to CON groups, although significantly higher miR-21, miR-192 and lower miR-200b expression was detected in the kidney cortex compared to the medulla (Figure 6C) regardless of the diet.

### 2.4. mRNA Expression of Inflammatory, Oxidative Stress, and Metabolic Markers in the Kidney in PRED Rats

**Figure 7:**
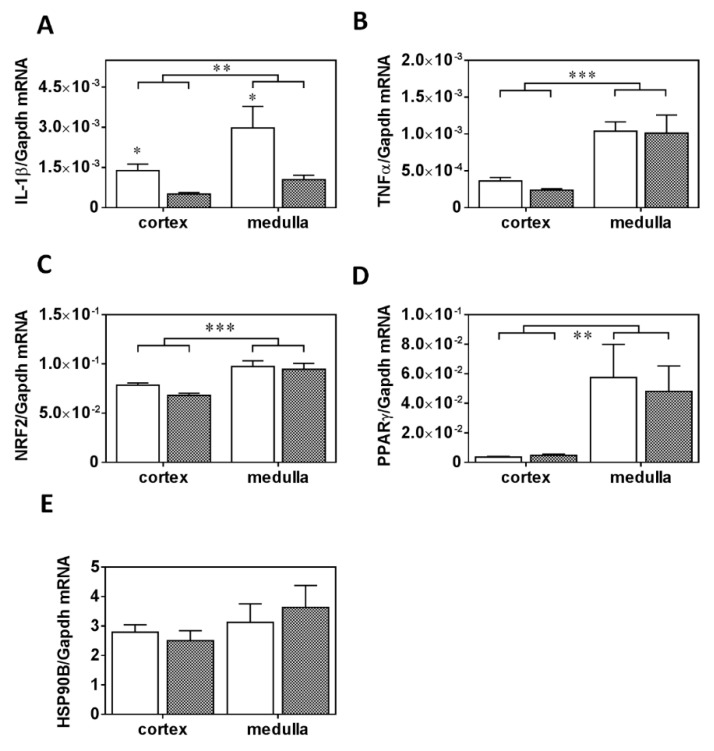
mRNA expression of inflammatory, oxidative stress and metabolic markers in the kidney (**A**) *IL-1β*, (**B**) *TNFα*, (C) *Nrf2*, (**D**) *PPARγ,* and (**E**) *HSP90B* mRNA expression in kidney cortex and medulla in obese prediabetic (PRED, gray columns) and control (CON, white columns) rats at the end of study. Data are means ± SEM, two-way RM ANOVA, n8-9/group (**A**–**E**); *: *p* < 0.05, **: *p* < 0.01; ***: *p* < 0.0001.

To our surprise, *IL-1β* mRNA expression was strongly reduced both in the kidney cortex and medulla (Figure 7A) in the PRED vs. the CON group, while *TNFα* mRNA expression was numerically (by about 40%) but insignificantly reduced only in the kidney cortex (Figure 7B). *Nrf2*, *PPARγ*, and *HSP90β* mRNA expression (Figure 7C–E) was not influenced in the PRED group. Marker genes of inflammation (*IL-1β, TNFα*), oxidative stress (*Nrf2*) and metabolic impairment (*PPARγ*) were typically expressed higher in the kidney medulla than in the cortex (Figure 7A–D).

### 2.5. Phosphorylation of Akt on Ser^473^ was Reduced in PRED Rat Livers

**Figure 8:**
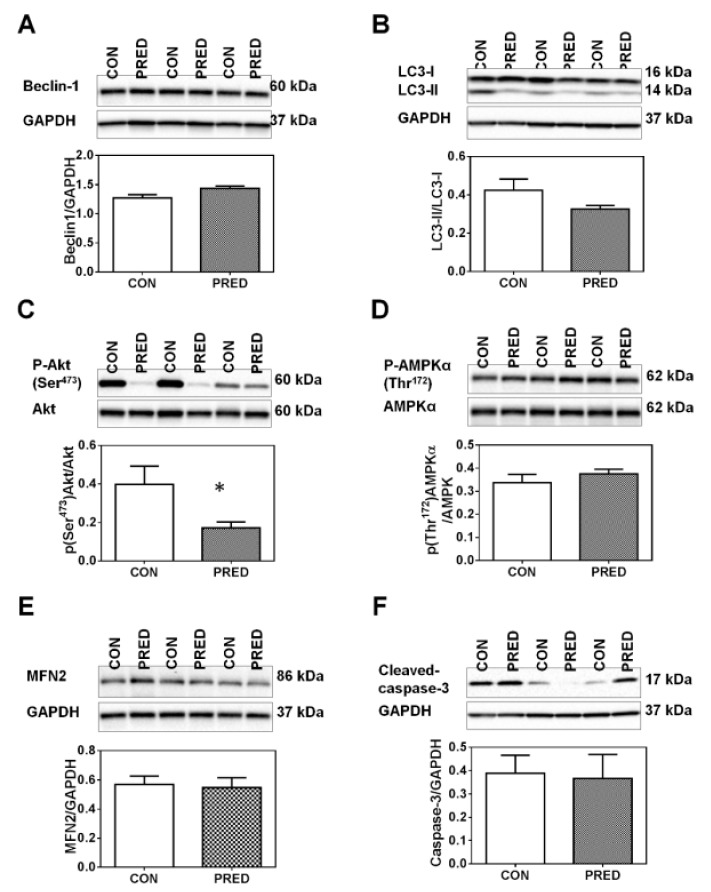
Protein phosphorylation and expression levels in the liver at the end of study. Representative western blot protein bands and relative protein expression of (**A**) Beclin-1, (**B**) *LC3*, phosphorylated (**C**) *Akt* and (**D**) *AMPK*, (**E**) and relative protein expression of *MFN2* and (**F**) cleaved-caspase-3 in liver samples of obese prediabetic (PRED, grey columns) and control (CON, white columns) rats at the end of the study. Data are means ± SEM, n = 8/group. Unpaired two-tailed Student’s t-test. *: *p* < 0.05.

Liver expression of autophagy-related proteins such as Beclin-1 and *LC3-II* was similar in the two groups (Figure 8A,B). *AMPKα* phosphorylation of *Akt* (an upstream modulator of autophagy) on Ser^473^ was reduced in the liver lysates (Figure 8C), while the phosphorylation of *AMPKα* on Thr^172^ was similar in the two groups (Figure 8D). Furthermore, the expression of a mitochondrial fusion-related protein *MFN2* (Figure 8E) and apoptosis-related cleaved-caspase-3 (Figure 8F) proteins was also similar in the two groups.

## 3. Discussion

The main finding of our study is that feeding Long-Evans rats with a HFD for 20 weeks and administering a single low dose of STZ at week 4 lead to elevated glomerular collagen deposition and caused tubular damage as demonstrated by increased cortical and medullary *Lcn2* mRNA expression but no other obvious kidney injury was observed. These mild renal effects were surprising as PRED rats had higher body weight, body fat content and insulin resistance. The results of the cardiovascular study performed in the same group of rats were published earlier [12]. Adipose tissue remodelling was also present in PRED rats as evidenced by increased plasma leptin concentration and *TGF-β1* mRNA in both the inguinal and epididymal adipose tissues, as well as by elevated *PPARγ* and adiponectin mRNA expression only in the epididymal adipose tissue. Furthermore, we had demonstrated hepatic steatosis, left ventricular diastolic dysfunction and hypertrophy in the same groups of PRED rats as published recently [12]. These results [12] collectively suggest that obesity with prediabetes caused organ injury that started earlier in the adipose tissue, heart, and liver than in the kidney.

Surprisingly, glomeruli of PRED rats had largely normal histomorphology. The average size of glomeruli did not increase, ruling out glomerulomegaly—a hallmark of ORG, at this stage of the disease. The relative PAS positive area of the glomerular tuft did not suggest accumulation of glomerular extracellular matrix despite significantly elevated glomerular collagen IV protein deposition in the glomerular basement membrane (GBM) of PRED rats. Accumulation of collagens, particularly types I, IVα3, and IVα4 in the GBM is a typical phenomenon both in a non-diabetic, HFD fed mouse model of obesity-related glomerulopathy (ORG) [13,14], and in a diabetic nephropathy model [15,16]. Increased glomerular collagen IV protein deposition can be the direct consequence of elevated plasma glucose in our study as high glucose leads to increased collagen IV synthesis in glomerular mesangial cells, in vitro [17]. Glomerular collagen IV protein deposition can be considered as a very early sign of ORG.

An important hallmark of ORG is glomerular hyperfiltration as a consequence of vasodilation of the afferent arteriole [18,19]. Preserved glomerular function, suggested by creatinine and urea levels falling into the normal range, did not support glomerular hyperfiltration in our PRED rats, nor did we observe any difference between the two groups in protein expression of desmin, an indicator of podocyte stress [20]. It seems contradictory that plasma urea levels, generally used to monitor renal excretory function, were decreased in PRED rats compared to that in CON animals. However, in cafeteria-diet fed rodents hepatic synthesis of urea significantly decreased due to limited availability of arginine [21,22]. Accordingly, lower plasma urea concentration in PRED rats can be due to decreased urea synthesis in the liver, and does not necessarily indicate increased glomerular filtration in PRED compared to CON animals in our study.

Increased cortical and medullary *Lcn2* mRNA expression demonstrated tubular injury in PRED rats. Thus, tubular injury also may be a very early event in ORG, later contributing to tubulointerstitial fibrosis and CKD progression. Furthermore, early *Lcn2* (*NGAL*) overproduction may accelerate CKD by increasing inflammation [23,24], apoptosis and decreasing cell proliferation [25,26]. Thus, the observed early *Lcn2* production may be a trigger of later progressive renal damage in obesity.

Among the inflammatory cytokines *IL-1β* represents a central mediator of inflammation in various tissues [27]. Surprisingly, both cortical and medullary *IL-1β* mRNA expression decreased in kidneys of PRED compared to that of CON rats. It has been demonstrated previously that increased renal and adipose tissue *TNFα* production is attributed to infiltrating pro-inflammatory macrophages contributing to obesity-related renal impairment [28,29,30]. However, *TNFα* mRNA expression was similar in the cortex and medulla in kidneys of PRED compared to that of CON rats. These results collectively suggest that there was less inflammation in the kidneys in our model. Furthermore, a somewhat decreased systemic inflammation was evident as plasma CRP levels decreased to a small extent in PRED vs. CON rats [12].

Leptin—an adipose tissue hormone correlates with the amount of body fat, therefore, obesity is accompanied with hyperleptinemia as also observed in our study [31]. The kidney expresses abundant amounts of the small isoform of the leptin receptor (Ob-Ra) [32,33] Leptin infusion upregulated glomerular *TGF-β1* and collagen type IV expression in rats [34]. Therefore, leptin can be an important contributor to obesity-induced kidney injury [35]. Plasma leptin increase was prominent in our study. Thus, we hypothesized that leptin/Ob-Ra/*TGF-β1* pathway could play a role in the elevated collagen type IV accumulation in GBM of glomeruli in the PRED group. However, *Ob-Ra* and *TGF-β1* expression were similar in PRED and CON kidneys. Thus, chronically high plasma leptin alone was not sufficient to induce ORG or renal tubulointerstitial fibrosis via Ob-Ra signalling in Long-Evans rats.

Although elevation of serum lipids (TG, total cholesterol, LDL, and HDL) was expected as a consequence of extreme obesity, serum lipid and liver enzyme concentrations were within the reference range and similar in PED and CON rats. There are some supporting results in the literature showing that HFD did not increase total cholesterol in all rat studies [36]. Furthermore, it is known that some obese patients have healthy lipid profile [37,38,39]. Thus, obesity likely leads to dyslipidemia, but not necessarily in all obese subjects. The observed resistance of the Long-Evans strain to obesity-related comorbidities is the most likely explanation for this lack of plasma lipid and liver-enzyme elevation. Therefore, our model offers a unique possibility to study the mechanisms of “obesity paradox” in Long-Evans rats modelling those patients who remain healthy despite obesity. Strain differences are not unusual in rats as we have shown previously concerning doxorubicin nephropathy.

Similarly to plasma lipids and liver enzymes, decreased plasma CRP concentration i.e., less systemic inflammation is unusual in obese models. To our current knowledge this is the first paper to report uncoupling excessive weight gain from inflammation. However, there is some supporting data in the literature reporting that few individuals with BMI close to or above 30 can have low CRP levels [40,41,42], thus, obesity does not necessarily cause inflammation in each individual. We hypothesize that different response to the adipose tissue expansion and/or missing inflammatory mediators may be responsible for this phenomenon.

The presence of intracellular lipid vacuoles is a characteristic finding in obesity both in rodent models and in the kidney of obese patients, suggesting that abnormal lipid metabolism and lipotoxicity may be the major cause of renal dysfunction [43,44,45]. In contrast to the heart and liver [12], renal intracellular lipid accumulation was undetectable in PAS or oil-red-O stained (missing microvacuoles) kidneys of PRED rats (see Supplementary Materials).

### Limitations of the Study

A limitation of this study is that it was performed exclusively on male rats. Disease processes can be substantially different in the two sexes (see NIH notice NOT-OD-15-102), e.g., female rats are less susceptible to kidney injury than males. Since only mild kidney injury could be demonstrated in male PRED rats it seems unlikely that we could draw a different conclusion in female rats.

Comparing our results to those published in the literature raises the question if Long-Evans rats are similarly sensitive to diet-induced obesity and prediabetes in comparison to other rat strains or HFD fed mouse models [46]. The majority of published results show that other rat strains develop ORG after 20 weeks or even after 10 weeks of HFD. To our best knowledge there are no available results to compare obesity-related co-morbidities in Long-Evans and other rat strains. Therefore, Long-Evans rats seem to be more resistant to ORG than other rat strains. However, the relative resistance of Long-Evans kidneys to obesity-related damage allowed us to study the order of injury development in various organs in PRED Long-Evans rats. Furthermore, the Long-Evans rat could be a good model of “obesity paradox”, as this strain can be used to identify the mechanisms of protection against obesity-related co-morbidities. Such information can have therapeutic utility in the future.

The results of this study demonstrated that long-term HFD-induced obesity combined with prediabetic metabolism was accompanied by collagen type IV accumulation in renal glomeruli and enhanced tubular *Lcn2 (NGAL)* production in Long-Evans rats, but otherwise renal function and morphology were preserved, while injury was observed in the heart and liver in the same animals. The relative resistance of Long-Evans strain to develop renal injury due to obesity and prediabetes is possibly attributable to reduced systemic and renal inflammation. The results seem to indicate that obesity may harm the liver and the heart earlier than the kidney in prediabetic Long-Evans rats fed a HFD. Thus, the Long-Evans rat strain may be suitable to study resistance mechanisms to obesity-related glomerulopathy.

## 4. Materials and Methods

### 4.1. Animal Model and Experimental Setup

#### 4.1.1. Ethics Statement

This investigation conforms to the Guide for the Care and Use of Laboratory Animals published by the US National Institutes of Health (Eighth Edition, 2011) and was approved (on 27. Dec. 2012.) by the Animal Ethics Committee of the Semmelweis University (registration number: XIV-I-001/2103-4/2012).

#### 4.1.2. Animal Model

Male Long-Evans rats aging 5–7 weeks were purchased from Charles River Laboratories (Wilmington, MA, USA). The animals were allowed free access to food and water ad libitum in a room maintained on a 12 h–12 h light/dark cycle and at a constant temperature of 21 °C. After 1 week of acclimatization the rats were divided into two groups: control (CON; n = 20) and prediabetic (PRED, n = 20). The control group was fed control chow, whereas the PRED group was fed a chow supplemented with 40% lard as a HFD [12]. To facilitate the development of prediabetes the animals on HFD received 20 mg/kg STZ (Santa Cruz Biotechnology, Dallas, TX, USA) intraperitoneally (i.p.) at the fourth week according to Mansor et al. [47], whereas the control group was treated with the same volume of citrate buffer as vehicle.

#### 4.1.3. Renal Sample Collection

At week 21 the animals were anesthetized with pentobarbital (60 mg/kg, i.p.; Euthasol; Produlab Pharma, Raamsdonksveer, The Netherlands) and underwent an extensive cardiac functional examination protocol (including echocardiography and hemodynamic analysis described and published earlier in detail [12]. From both groups 9 randomly selected rats were used to evaluate the renal effects of HFD. Blood samples were collected by puncture of the abdominal aorta into EDTA-prefilled blood collection tubes and the heart was removed in toto. Immediately upon these procedures the thoracic aorta was ligated, and the carcass was perfused with 50–80 mL ice-cold physiological saline via an aortic cannula inserted below the origin of the renal arteries. Application of sodium heparin or other systemic anticoagulant was avoided as it was not allowed in the cardiac protocol. Eye-control assured complete blood flush from the liver and the kidneys. The kidneys, liver, epididymal, and subcutaneous, inguinal white adipose tissues were removed and weighed on an analytical balance. Small parts of the liver and a 1–2 mm horizontal sections of the left kidney were fixed in 4% buffered formaldehyde for 24 h, and then were dehydrated and embedded in paraffin wax (FFPE) for histology and immunohistochemistry. Similar pieces were embedded in Tissue Tek O.C.T. Compound (Sakura Finetech, Europe) and were slowly frozen on dry ice for analysis of fat deposition. Kidney cortex and medulla samples from the right kidney were separated by sterile surgical scalpel, sufficient pieces of liver and adipose tissue samples were snap-frozen in liquid nitrogen in cryotubes and stored at –80°C for molecular studies.

### 4.2. Analysis of Functional Kidney Parameters

Blood plasma was separated by centrifugation at 5000 rpf for 10 min, at 4 °C. Urine samples were centrifuged at 5000× *g* for 5 min at 4 °C to remove the sediment, and then were stored at –80 °C until analysis.

Plasma carbamide, plasma and urine creatinine concentrations were assessed using a colorimetric, enzymatic assay (#9581C and #9571C respectively; Diagnosticum Ltd. Budapest, Hungary) in 96 well plates (Greiner Bio-One GmbH, Frickenhausen, Germany).

Urine total protein concentration was assessed using a pyrogallol red colorimetric assay (#42051/DC, Diagnosticum Ltd., Budapest, Hungary). The results were normalised to urine creatinine concentration. Optical densities were measured at 598 nm (protein assay) and 555 nm (creatinine assay) in a SpectraMax 340 Microplate Reader (MolecularDevices, Sunnyvale, CA, USA). Concentrations were calculated using SoftMax^®^ Pro Software (Molecular Devices, Sunnyvale, CA, USA).

Plasma and urine lipocalin-2 (*Lcn2*) concentrations were measured using rat *Lcn2/NGAL* DuoSet ELISA Development kit (R&D Systems, Minneapolis, MN, USA). Optical density was measured at 450 nm with wavelength correction set at 544 nm using Victor3 1420 Multilabel Counter (PerkinElmer, WALLAC Oy, Turku, Finland). Concentrations were calculated using a four-parameter logistic curve-fit (Work Out; Dazdaq Ltd., Brighton, England).

### 4.3. Analysis of Renal Morphology

Routine histological and immunohistochemical analysis was performed on FFPE tissue samples. Alterations in glomerular or tubulointerstitial morphology were evaluated in sections stained with periodic-acid-Schiff (PAS).

### 4.4. Analysis of Plasma Lipid and Functional Liver Parameters

Plasma cholesterol, low density lipoprotein (LDL), high-density lipoprotein (HDL), triglyceride, glutamate oxaloacetate transaminase (GOT), and glutamate pyruvate transaminase (GPT) were measured by automated clinical laboratory methods (Diagnosticum, Budapest, Hungary). Plasma leptin concentration was measured by ELISA (Invitrogen, Camarillo, CA, USA).

### 4.5. Immunohistochemistry

Paraffin sections mounted on Superfrost Ultra Plus Adhesion Slides (Thermo Fisher Scientific Inc, Waltham, MA, USA) were deparaffinized and rehydrated in ethanol. Desmin, αSMA and fibronectin immunohistochemistry was performed with polyclonal antibodies (anti-desmin MS 376-S1, Thermo Fisher, 1:1000; anti- αSMA ab5694, Abcam, 1:1000; anti-FN HPA0027066, Atlas Antibodies, 1:1000), using the avidin–biotin method and developed by incubation with diaminobenzidine (Vector Laboratories, Burlingame, CA, USA). Pictures were taken with Zeiss AxioCam 512 of the stained sections for further analysis. Glomerular tuft was delineated manually and standard glomerulus size parameters (e.g., Feret diameter) as wells as the PAS, desmin, and αSMA positive area were determined using the CellProfiler cell image analysis software.

Lipid deposition was determined by oil-Red-O (O0625, Sigma-Aldrich, Budapest, Hungary) staining of 5-μm-thick cryosections.

### 4.6. Gene-Expression Analysis of the Renal and Adipose Tissue Samples

#### 4.6.1. RNA Preparation

Total RNA was extracted from the snap-frozen tissue samples (kidney, liver, adipose tissues) with TRI Reagent^®^ (Molecular Research Center, Inc., Cincinnati, OH, USA) according to the protocol provided by the manufacturer [48]. In brief, the frozen renal tissues were homogenized by an IKA^®^ DI 18 basic grinder (IKA^®^ Works do Brasil Ltd.a., Taquora, Brazil). Chloroform (Sigma-Aldrich, Inc., St Louis, MO, USA) was added to each sample and mixed by vortexing. The aqueous phase was separated from the organic phase by centrifugation. RNA was precipitated from the transferred aqueous phase with an equal quantity of isopropyl alcohol by incubation for 30 min at room temperature. The RNA pellet was washed twice with 75% ethyl alcohol, and dissolved in 100 μL RNase free water. The RNA concentration and purity was assessed with a NanoDrop 2000c Spectrophotometer (Thermo Fisher Scientific, Wilmington, DE, USA). All RNA samples had an absorbance ratio (260 nm/280 nm) above 1.8. To check RNA integrity, the samples were electrophoresed on 1% agarose gel (Invitrogen Ltd., Paisley, UK) in BioRad Wide mini-sub^®^ cell GT system (Bio-Rad Laboratories, Inc., Hercules, CA, USA), and the ratio of 28S ribosomal RNA bands was calculated. The RNA solutions were kept at −80 °C until further procedures.

#### 4.6.2. Quantitative Real-Time PCR Analysis

Messenger RNA and miRNA levels in the kidney cortex or medulla and epididymal or inguinal white adipose tissue samples were measured by double-stranded DNA (dsDNA) dye based real-time PCR using Bio-Rad CFX96 Real Time System with a C1000 Thermal Cycler. Results were calculated with the relative quantification (ΔΔCq) method, and the efficiency of the quantitative PCR reaction was verified with standard curves.

#### 4.6.3. Messenger RNA Detection

First, reverse transcription of 1 μg total renal RNA into cDNA was carried out using random hexamer primers and the High-Capacity cDNA Archive Kit (Applied Biosystem, USA) according to the manufacturer’s protocol in Bio Rad iCycler™ Thermal Cycler (Bio-Rad Laboratories, Inc., Hercules, CA, USA). Second, in the real-time polymerase chain reaction (PCR) step, PCR products were amplified from the cDNA samples using SensiFAST™ SYBR^®^ No-ROX Master Mix (Bioline) and target specific primer pairs to detect messenger RNA levels of adiponectin (*Adpn*), interleukine-1 beta (*IL-1β*), leptin receptor short-form (*Ob-Ra*), neutrophil gelatinase associated *Lcn2* (*NGAL*), nuclear factor (erythroid-derived 2)-like 2 (*NFE2L2* or *Nrf2*), *PPAR*-gamma (*PPARγ*), tumor necrosis factor alpha (*TNFα*), transforming growth factor β1 (*TGF-β1*) were used. Primers were self-designed by the NCBI/Primer-BLAST online software and synthesized by Integrated DNA Technologies (IDT, Inc., Coralville, IA, USA), for detailed list of Fwd and Rev primer sequences see Table 1). All measurements were done in duplicates. Target mRNA levels were normalized to *Gapdh* mRNA or to 28S rRNA levels.

#### 4.6.4. microRNA Detection: 

Expression of microRNAs was evaluated with TaqMan probes (Chen et al., 2011). First, complementary DNA (cDNA) was reverse-transcribed (RT) from 5 ng RNA sample using a miRNA-specific, stem-loop RT primer (for miR-21, miR-29b, miR-192, miR-200a, miR-200b, and U6 snRNA) from the TaqMan^®^ Small RNA Assays and reagents from the TaqMan^®^ MicroRNA Reverse Transcription Kit (Applied Biosystems™), as described in the manufacturer’s protocol. Second, in the real-time polymerase chain reaction (PCR) step, PCR products were amplified from the cDNA samples using the TaqMan^®^ Small RNA Assay together with the TaqMan^®^ Universal PCR Master Mix 2. All measurements were done in duplicates, and the miRNA expressions were normalized to the U6 small nuclear RNA (snRNA) applied as an endogenous reference [49]. Since U6 expression levels were found regulated in our model, the target microRNA levels were also normalized to the median of all miRNA measurements Sq values, which didn’t reveal major differences in the final results.

### 4.7. Western Blot of Liver Lysates

Freeze clamped liver samples were pulverized under liquid nitrogen and homogenized in homogenization buffer containing (in mmol/L): 20 Tris-HCl, 250 sucrose, 1.0 EGTA, 1.0 dithiothreitol, or in radioimmunoprecipitation assay buffer (Cell Signaling Technology, Danvers, MA, USA), supplemented with 1 mM phenylmethylsulphonylfluoride (PMSF; Roche, Basel, Switzerland), 0.1 mM sodium fluoride, 200mM sodium orthovanadate and complete protease inhibitor cocktail (Roche) with TissueLyser LT (Qiagen, Venlo, The Netherlands) to obtain liver whole cell lysate. Protein samples were resolved on precast 4%–20% Criterion TGX gels (Bio-Rad, Hercules, CA, USA) and transferred to nitrocellulose or Immun-Blot PVDF membranes (Bio-Rad). Quality of transfer was verified with Ponceau S staining. Membranes were blocked with 5% nonfat milk (Bio-Rad) or 2%–5% bovine serum albumin (BSA; Santa Cruz Biotechnology, Dallas, TX, USA) in Tris-buffered saline with 0.05% Tween 20 (TBS-T) for 0.5-2 h. Membranes were incubated with primary antibodies in 1%–5% nonfat milk or BSA in TBS-T: anti-caspase-3 (1:500; Santa Cruz Biotechnology), anti-mitofusin-2 (*MFN2*; 1:2500, Abcam, Cambridge, UK), anti-microtubule-associated protein 1 light chain 3 A/B (*LC3* A/B; 1:5000), anti-Beclin-1 (1:1000), anti-phospo-*Akt* (Ser^473^; 1:1000), anti-*Akt* (1:1000), anti-phospho-*AMP*-activated protein kinase α (*AMPKα*-Thr^172^; 1:1000), anti-*AMPKα* (1:1000), and anti-*GAPDH* (1:5000) as loading control (Cell Signaling Technology). After three washes with TBS-T, horseradish peroxidase conjugated secondary antibody was added for 2 h at room temperature (1:5000 in 5% nonfat milk in TBS-T). Signals were detected with an enhanced chemiluminescence kit (Bio-Rad, Hercules, CA) by Chemidoc XRS+ (Bio-Rad). Antibodies against phosphorylated epitopes were removed with Pierce Stripping Buffer (Thermo Fisher Scientific, Waltham, MA, USA) before incubation with antibodies detecting the total protein.

### 4.8. Statistical Analysis

The results are presented as mean ± standard error of the mean (SEM) unless otherwise indicated. Logarithmic transformation was performed if Bartlett’s test indicated inhomogeneity of variances. Continuous variables were compared using either Student’s unpaired “t” test or two-way ANOVA with Tukey’s multiple comparisons test. Body weight gain was analysed using two-way ANOVA for repeated measurements (two-way RM ANOVA) followed by Sidak’s post hoc test. The null-hypothesis was rejected if the p value reached statistical significance (*: *p* < 0.05, **: *p* < 0.01, ***: *p* < 0.001). GraphPad Prism6 software (GraphPad Software, La Jolla, CA, USA) was used for data management, statistical analysis and depicting figures.

## Figures and Tables

**Table 1:** qPCR Primer Sequences.

Species	Gene Symbol	Primer Direction	Sequence (5′->3′)
rno	*Adpn*	fwd	5′-AAAGGAGAGCCTGGAGAAGC-3′
		rev	5′-GTCCCGGAATGTTGCAGTAG-3′
rno	*Gapdh*	fwd	5′-AACTTGCCGTGGGTAGAG-3′
		rev	5′-ATGGTGAAGGTCGGTGTG-3′
rno	*IL-1* *β*	fwd	5′-AGA GTG TGG ATC CCA AAC AA -3′
		rev	5′-AGT CAA CTA TGT CCC GAC CA -3′
rno	*Lcn2*	fwd	5′-GATGTTGTTATCCTTGAGGCCC-3′
		rev	5′-CACTGACTACGACCAGTTTGCC-3′
rno	*Nrf2*	fwd	5′-CTC TCT GGA GAC GGC CAT GAC T-3′
		rev	5′-CTG GGC TGG GGA CAG TGG TAG T-3′
rno	*Ob-Ra/532*	fwd	5′-GACGATGTTCCAAACCCCAAG-3′
		rev	5′-TGGGAGGTTGGTAGATTGGATTC-3′
rno	*PPARγ*	fwd	5′-CTGCCTATGAGCACTTCACAAG-3′,
		rev	5′-ATCACGGAGAGGTCCACAGA-3′
rno	*TGF-* *β* *1*	fwd	5′-AGCCCTGTATTCCGTCTCCT-3′
		rev	5′-ATTCCTGGCGTTACCTTGG-3′
rno	*TNFα*	fwd	5′-TTCTCATTCCTGCTCGTGGC-3′
		rev	5′-AACTGATGAGAGGGAGCCCA-3′
rno	*HSP90B*	fwd	5′-GGAAGCCCCCGCCCTCTGTATA-3′
		rev	5′-AGGGCCAGTCAAGGCTGTTGG-3′
rno	28S	fwd	5′-GGTAAACGGCGGGAGTAACT-3′
		rev	5′-TCACCGTGCCAGACTAGAGT-3′

## Supplementary Materials

Supplementary materials can be found at https://www.mdpi.com/1422-0067/20/17/4266/s1.
