# Supplementary figures and images for: Glomerular Collagen Deposition and Lipocalin-2 Expression Are Early Signs of Renal Injury in Prediabetic Obese Rats
Source: Int J Mol Sci. 2019 Aug 30;20(17):4266. doi: 10.3390/ijms20174266 (PMC6747173; doi:10.3390/ijms20174266)

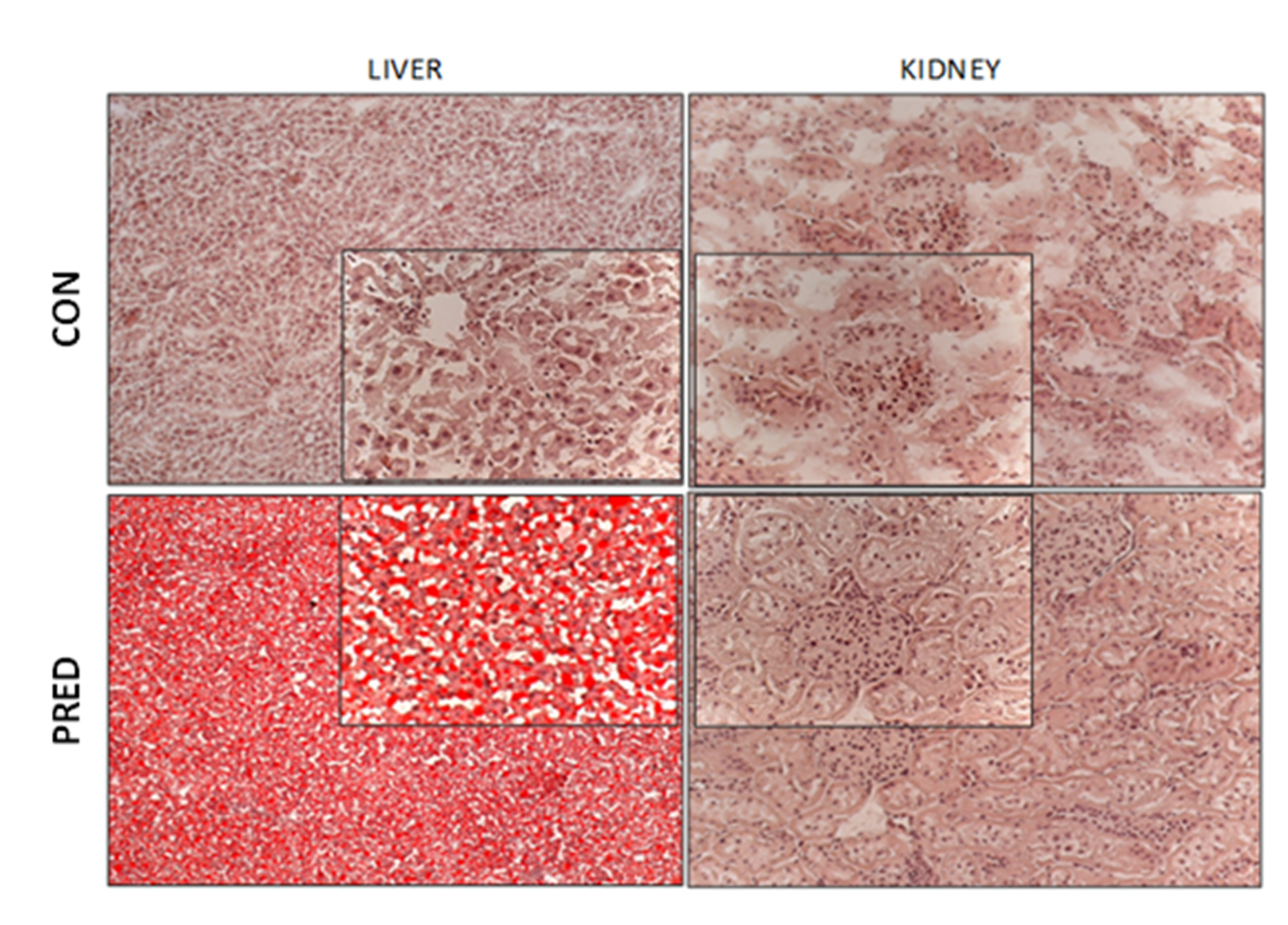

Supplement: Supplementary file 1 [file ijms-20-04266-s001.zip › ijms-581845 supplementary/suppl fig 1 IJMS R.tif]
